# Outcomes and Prognostic Factors of Cetuximab-Based Therapy in Recurrent/Metastatic Head and Neck Squamous Cell Carcinoma

**DOI:** 10.3390/jcm15155852

**Published:** 2026-07-27

**Authors:** Melike Dönmez Tekin, Atike Pınar Erdoğan, Mustafa Şahbazlar, Ferhat Ekinci

**Affiliations:** Department of Medical Oncology, Faculty of Medicine, Manisa Celal Bayar University, Manisa 45030, Turkey

**Keywords:** head and neck squamous cell carcinoma, cetuximab, overall survival, prognostic factors

## Abstract

**Background:** Cetuximab-based therapies continue to play an important role in the management of recurrent/metastatic head and neck squamous cell carcinoma (R/M HNSCC) despite the increasing use of immunotherapy. However, data regarding treatment outcomes and prognostic factors remain limited. This study aimed to evaluate treatment outcomes and prognostic factors associated with survival in patients with R/M HNSCC treated with cetuximab-based regimens. **Methods:** This retrospective single-center cohort study included patients with recurrent/metastatic HNSCC who received cetuximab-based systemic therapy at the Department of Medical Oncology, Manisa Celal Bayar University Faculty of Medicine, between May 2012 and September 2025. Demographic, clinical, laboratory, and PET/CT data were retrospectively analyzed. Progression-free survival (PFS) and overall survival (OS) were estimated using the Kaplan–Meier method and compared using the log-rank test. Prognostic factors associated with survival were evaluated using univariable and multivariable Cox proportional hazards regression analyses. **Results:** A total of 57 patients were included in the study. The median age was 65 years, and 91.2% of patients were male. Most patients had ECOG performance status 0–1 (94.7%) and stage IV disease (71.9%). The larynx was the most common primary tumor site (50.9%), while distant lymph node metastasis (63.2%) and lung metastasis (61.4%) were the predominant metastatic sites. Median PFS and OS were 4.6 months and 13.8 months, respectively. Kaplan–Meier analysis demonstrated that patients with a body mass index (BMI) > 25 had longer OS, whereas chronic kidney disease (CKD), primary tumor localization, and best response to first-line treatment were significantly associated with survival. In multivariable Cox regression analysis, chronic kidney disease (CKD) (HR: 5.15, *p* = 0.004), oral cavity primary tumor localization (HR: 2.55, *p* = 0.013), progressive disease as the best response to first-line treatment (HR: 4.44, *p* < 0.001) and the absence of grade 1–2 cetuximab-related dermatologic toxicity (HR: 3.20, *p* = 0.027) remained independent adverse prognostic factors. BMI was not independently associated with survival in multivariable analysis. **Conclusions:** Cetuximab-based therapy demonstrated clinically meaningful activity in patients with R/M HNSCC. Comorbidities, primary tumor localization, the absence of grade 1–2 cetuximab-related dermatologic toxicity and treatment response appear to substantially influence survival outcomes. Larger prospective studies are warranted to validate these findings.

## 1. Introduction

Head and neck squamous cell carcinoma (HNSCC) is one of the most aggressive epithelial malignancies worldwide and remains associated with substantial morbidity and mortality despite advances in multidisciplinary treatment strategies. Tobacco exposure, alcohol consumption, and human papillomavirus (HPV)-related carcinogenesis are among the major etiological factors contributing to disease development. Although many patients present with locoregionally advanced disease at diagnosis, a considerable proportion eventually develop recurrent or metastatic disease during follow-up, resulting in limited survival outcomes and a substantial treatment burden [1].

The prognosis of recurrent/metastatic HNSCC (R/M HNSCC) remains poor, with historically reported median overall survival ranging between 6 and 12 months. For many years, cetuximab-based treatment represented a cornerstone of first-line systemic therapy in R/M HNSCC following the landmark EXTREME trial, which demonstrated superior survival outcomes with the addition of cetuximab to platinum-based chemotherapy and 5-fluorouracil compared with chemotherapy alone [2]. More recently, immune checkpoint inhibitors, particularly pembrolizumab and nivolumab, have substantially changed the therapeutic landscape of R/M HNSCC and improved survival outcomes in selected patient populations [3,4].

Nevertheless, cetuximab-containing regimens continue to retain an important role in routine oncology practice. In particular, these regimens remain valuable for patients with rapidly progressive symptomatic disease requiring prompt tumor shrinkage, contraindications to immunotherapy, low or unavailable programmed death-ligand 1 (PD-L1) expression, or poor suitability for immunotherapy-based approaches. Furthermore, treatment patterns frequently differ from those observed in highly selected clinical trial populations. However, data regarding cetuximab-based treatment outcomes and prognostic factors in R/M HNSCC remain limited [5].

Identification of clinically applicable prognostic factors may contribute to treatment stratification and optimization of patient selection in daily oncology practice. Clinical variables, nutritional status, comorbid conditions, inflammatory biomarkers, and treatment-related factors have been investigated across different malignancies; however, their prognostic significance in cetuximab-treated R/M HNSCC populations remains incompletely understood [6,7]. Therefore, the present study aimed to evaluate treatment outcomes and prognostic factors associated with survival in patients with recurrent/metastatic HNSCC treated with cetuximab-based therapy.

## 2. Materials and Methods

### 2.1. Study Design and Patient Population

This retrospective single-center cohort study included patients diagnosed with recurrent and/or metastatic head and neck squamous cell carcinoma (R/M HNSCC) who received cetuximab-based systemic therapy at the Department of Medical Oncology, Manisa Celal Bayar University Faculty of Medicine, between May 2012 and September 2025.

Patients were eligible for inclusion if they were aged 18 years or older, had histopathologically confirmed head and neck squamous cell carcinoma, subsequently developed recurrent and/or metastatic disease, received at least one cycle of cetuximab-based systemic therapy for recurrent/metastatic disease, and had complete clinical, laboratory, treatment, and follow-up data available.

Some patients had received induction chemotherapy during the earlier course of their disease. However, only patients who subsequently developed recurrent and/or metastatic disease and received cetuximab-based systemic therapy in the recurrent/metastatic setting were included in the present study. Therefore, all treatment outcome and survival analyses were performed exclusively in the recurrent/metastatic setting.

Patients with non-squamous histology, nasopharyngeal carcinoma, or incomplete medical records were excluded from the study.

The study was conducted in accordance with the ethical principles of the Declaration of Helsinki and approved by the Manisa Celal Bayar University Faculty of Medicine Health Sciences Ethics Committee (decision no: 20.478.486/3530; date: 5 November 2025).

### 2.2. Data Collection and Clinical Variables

Clinical, demographic, laboratory, and imaging data were retrospectively collected from electronic medical records. Recorded clinical and demographic variables included age, sex, height, weight, body mass index (BMI), Eastern Cooperative Oncology Group (ECOG) performance status, comorbid diseases, tumor presentation (de novo metastatic or recurrent disease), primary tumor localization, and sites of metastatic involvement.

Pretreatment laboratory parameters, including serum albumin, C-reactive protein (CRP), lactate dehydrogenase (LDH), hemoglobin, platelet count, neutrophil count, lymphocyte count, monocyte count, creatinine, glomerular filtration rate (GFR), alanine aminotransferase (ALT), total bilirubin, and alkaline phosphatase (ALP), were recorded for all patients.

Pretreatment fluorodeoxyglucose positron emission tomography/computed tomography (FDG PET/CT) findings were retrospectively reviewed, and the maximum standardized uptake value (SUVmax) of both primary tumors and metastatic lesions was documented for further analyses.

The Advanced Lung Cancer Inflammation Index (ALI) was calculated to evaluate its potential prognostic significance. ALI was calculated using the following formula: BMI × serum albumin/neutrophil-to-lymphocyte ratio (NLR).

### 2.3. Treatment Characteristics

Treatment response was radiologically evaluated using contrast-enhanced computed tomography during routine clinical follow-up. Best overall response was categorized as complete response (CR), partial response (PR), stable disease (SD), or progressive disease (PD) based on radiological assessment.

Cetuximab-related adverse events were retrospectively evaluated using patient medical records. Dermatologic toxicities, including acneiform rash associated with epidermal growth factor receptor (EGFR) inhibition, were documented.

Treatment-related toxicities were graded according to the Common Terminology Criteria for Adverse Events (CTCAE), version 5.0.

### 2.4. Survival Definitions

Progression-free survival (PFS) was defined as the time interval between initiation of cetuximab-based therapy and the first documented radiological disease progression or death from any cause, whichever occurred first.

Overall survival (OS) was defined as the time interval between treatment initiation and death from any cause or the date of last follow-up.

Patients who remained alive without documented disease progression at the time of analysis were censored at the date of last follow-up.

### 2.5. Statistical Analysis

All statistical analyses were performed using IBM SPSS Statistics version 27.0 (IBM Corp., Armonk, NY, USA).

Continuous variables were expressed as mean ± standard deviation (SD) or median (range), as appropriate, whereas categorical variables were presented as frequencies and percentages.

Data distribution was assessed using skewness and kurtosis analyses. Comparisons between two groups were performed using the independent samples *t*-test or Mann–Whitney U test, whereas comparisons among multiple groups were conducted using one-way analysis of variance (ANOVA) or the Kruskal–Wallis test, as appropriate. Categorical variables were compared using the chi-square test or Fisher’s exact test.

Survival analyses were performed using the Kaplan–Meier method and compared using the log-rank test. Prognostic factors associated with overall survival (OS) were evaluated using univariable and multivariable Cox proportional hazards regression analyses. To minimize the risk of overfitting, only variables that were statistically significant in the univariable analyses (*p* < 0.05) or considered clinically relevant were included in the multivariable Cox regression model. All statistical tests were two-sided, and a *p*-value < 0.05 was considered statistically significant.

## 3. Results

### 3.1. Patient Characteristics

A total of 57 patients were included in the study. Baseline demographic and clinicopathological characteristics of the study population are summarized in Table 1.

The median age was 65 years (range, 41–81 years), and the majority of patients were male (91.2%). Most patients had a good performance status, with an ECOG performance status of 0–1 in 94.7% of the cohort.

Regarding disease presentation, 54.4% of patients presented with de novo metastatic disease, whereas 45.6% developed recurrent disease following prior treatment.

The larynx was the most common primary tumor site (50.9%), followed by the oral cavity (19.3%), hypopharynx (10.5%), and oropharynx (3.5%). The most frequent metastatic sites were distant lymph nodes metastases (63.2%) and the lungs metastases (61.4%).

Among comorbid conditions, hypertension (28.1%), coronary artery disease (21.1%), diabetes mellitus (15.8%), and chronic kidney disease (12.3%) were the most commonly observed.

### 3.2. Treatment Characteristics

Treatment-related characteristics are summarized in Table 2.

A previous history of induction chemotherapy was present in 10.5% of patients, whereas 43.9% had a history of prior curative radiotherapy and 17.5% had undergone surgery.

Cetuximab combined with platinum and 5-fluorouracil represented the predominant first-line treatment strategy. Specifically, 53.7% of patients received cetuximab plus cisplatin and 5-fluorouracil, while 13.0% received cetuximab plus carboplatin and 5-fluorouracil. Other cetuximab-containing regimens were administered in 33.3% of patients.

Regarding treatment response, the best response to first-line treatment consisted of complete response (CR) in 9.3%, partial response (PR) in 29.6%, stable disease (SD) in 25.9%, and progressive disease (PD) in 35.2% of patients.

Grade 1–2 cetuximab-related adverse events, consisting exclusively of dermatologic toxicity (acneiform rash), were observed in 19.3% of patients.

Regarding subsequent treatment, 36.8% of patients received nivolumab as second-line therapy, whereas 12.3% underwent third-line treatment.

### 3.3. Survival Outcomes

At the time of analysis, 50 deaths occurred during follow-up. The median progression-free survival (PFS) for the entire cohort was 4.6 months, whereas the median overall survival (OS) was 13.8 months. Kaplan–Meier survival analyses according to significant clinicopathological and treatment-related variables are summarized in Table 3.

**Figure 1:**
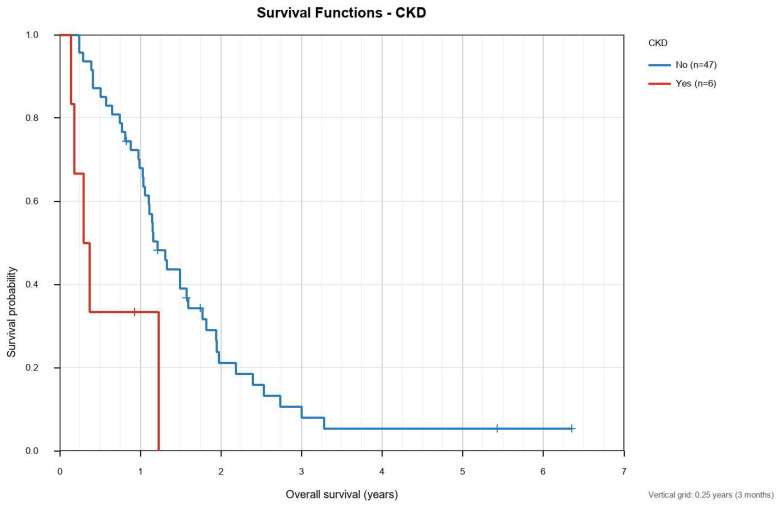
Kaplan–Meier curve for overall survival according to chronic kidney disease (CKD) status. Patients with CKD demonstrated significantly inferior survival outcomes compared with those without CKD (median OS: 3.5 vs. 14.6 months, log-rank *p* = 0.003).

Patients with a BMI > 25 kg/m^2^ demonstrated significantly prolonged OS compared with those with BMI ≤ 25 kg/m^2^ (median OS: 19.1 vs. 11.9 months, *p* = 0.002). Similarly, the presence of chronic kidney disease (CKD) was associated with markedly inferior survival outcomes, with a median OS of 3.5 months compared with 14.6 months in patients without CKD (*p* = 0.003, Figure 1).

**Figure 2:**
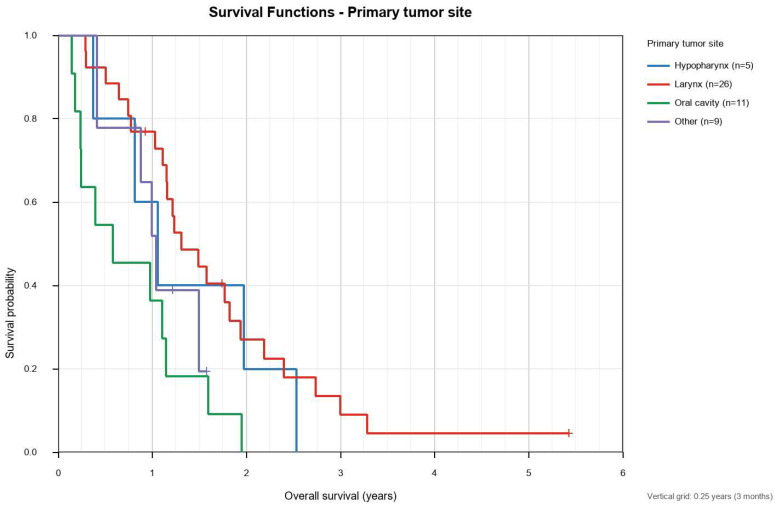
Kaplan–Meier curve for overall survival according to primary tumor site. Patients with laryngeal tumors demonstrated the most favorable survival outcomes, whereas oral cavity tumors were associated with poorer prognosis (log-rank *p* = 0.027).

Survival outcomes also differed according to primary tumor localization (*p* = 0.027). Patients with laryngeal tumors demonstrated the most favorable prognosis (median OS: 15.7 months), whereas oral cavity tumors were associated with poorer survival outcomes (median OS: 6.9 months, Figure 2).

Best response to first-line treatment was significantly associated with survival (*p* < 0.001). Patients achieving complete response (CR) demonstrated the most favorable outcomes (median OS: 23.7 months), whereas those with progressive disease (PD) had the poorest prognosis (median OS: 5.0 months).

**Figure 3:**
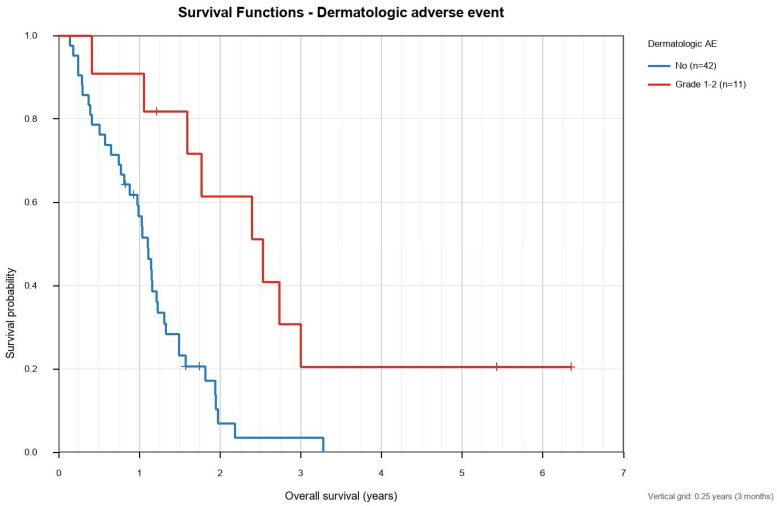
Kaplan–Meier curve for overall survival according to cetuximab-related dermatologic toxicity. Patients who developed grade 1–2 acneiform rash demonstrated significantly prolonged survival compared with those without dermatologic toxicity (median OS: 30.4 vs. 13.2 months, log-rank *p* < 0.001).

In addition, patients who developed cetuximab-related dermatologic toxicity (grade 1–2 acneiform rash) demonstrated significantly prolonged survival compared with those without toxicity (median OS: 30.4 vs. 13.2 months, *p* < 0.001, Figure 3).

### 3.4. Cox Regression Analysis

Univariable and multivariable Cox proportional hazards regression analyses for overall survival are summarized in Table 4.

In the univariable analysis, BMI, chronic kidney disease (CKD), primary tumor site, best response to first-line treatment, and the absence of grade 1–2 cetuximab-related dermatologic toxicity were significantly associated with overall survival (OS).

In the multivariable Cox regression analysis, chronic kidney disease remained an independent adverse prognostic factor for OS (HR: 5.15, 95% CI: 1.69–15.64, *p* = 0.004). Likewise, oral cavity localization of the primary tumor was independently associated with poorer survival (HR: 2.55, 95% CI: 1.22–5.34, *p* = 0.013). Progressive disease (PD) as the best response to first-line treatment also remained independently associated with inferior OS (HR: 4.44, 95% CI: 2.14–9.21, *p* < 0.001). In addition, the absence of grade 1–2 cetuximab-related dermatologic toxicity was independently associated with worse survival (HR: 3.20, 95% CI: 1.15–8.96, *p* = 0.027).

Although BMI was significantly associated with OS in the univariable analysis, it did not retain statistical significance in the multivariable model (*p* = 0.201).

## 4. Discussion

In this retrospective cohort of patients with recurrent or metastatic head and neck squamous cell carcinoma (R/M HNSCC) treated with cetuximab-based therapy, the median PFS and OS were 4.6 months and 13.8 months, respectively. Several clinicopathological and treatment-related factors were associated with survival outcomes. In multivariable analysis, chronic kidney disease (CKD), oral cavity localization of the primary tumor, and progressive disease (PD) as the best response to first-line treatment, and the absence of grade 1–2 cetuximab-related dermatologic toxicity emerged as independent prognostic factors for overall survival. These findings emphasize the importance of both baseline patient-related characteristics and treatment-related factors in determining outcomes among patients receiving cetuximab in routine clinical practice.

The survival outcomes observed in our cohort were generally consistent with previously reported clinical trial and retrospective cohort studies evaluating cetuximab-based treatment in R/M HNSCC. In the landmark EXTREME trial, the addition of cetuximab to platinum-based chemotherapy significantly improved survival, with a reported median OS of 10.1 months and median PFS of 5.6 months in patients with recurrent or metastatic disease [2]. Although direct comparisons should be interpreted cautiously because of differences in patient selection, performance status, and treatment heterogeneity, the median OS observed in our study (13.8 months) appears comparable or slightly more favorable than historical outcomes. This difference may partly reflect improvements in supportive care, patient selection, and access to subsequent treatment lines in routine clinical practice. Furthermore, outcomes from observational studies and contemporary treatment cohorts suggest that survival in R/M HNSCC remains heterogeneous and strongly influenced by baseline patient characteristics, treatment response, and access to subsequent therapies [3,5].

One of the most notable findings of our study was the strong negative prognostic impact of chronic kidney disease (CKD), which remained independently associated with inferior OS in multivariable analysis. Patients with CKD demonstrated substantially inferior survival outcomes and a markedly increased mortality risk. Although CKD has not been extensively evaluated as an isolated prognostic factor in cetuximab-treated R/M HNSCC populations, impaired renal function is known to adversely influence treatment tolerance, eligibility for platinum-based chemotherapy, and overall physiological reserve [5,8]. In particular, renal dysfunction may limit the use of cisplatin-containing regimens, increase susceptibility to treatment-related complications, and reflect a greater burden of comorbidity, all of which may contribute to poorer oncologic outcomes [9].

Another important finding was the independent association between oral cavity localization and poorer survival outcomes. Compared with other primary tumor sites, patients with oral cavity tumors demonstrated significantly inferior prognosis in multivariable analysis. This observation may be explained by the distinct biological behavior of oral cavity squamous cell carcinoma, which is often characterized by a more aggressive clinical course, higher rates of locoregional recurrence, and lower responsiveness to systemic treatment in the recurrent/metastatic setting [1,10]. In addition, unlike oropharyngeal tumors, oral cavity cancers are generally HPV-negative, a factor consistently associated with less favorable survival outcomes [11].

In the present study, cetuximab-related grade 1–2 dermatologic toxicity (acneiform rash) was associated with prolonged OS in univariable analyses. Patients who developed dermatologic toxicity demonstrated improved survival compared with those without toxicity. This finding is consistent with previous reports suggesting that EGFR inhibitor-related skin toxicity may reflect stronger pharmacodynamic EGFR blockade and serve as a surrogate marker of treatment efficacy [2,12]. Importantly, the absence of grade 1–2 cetuximab-related dermatologic toxicity remained an independent adverse prognostic factor in the multivariable analysis. This finding further supports previous observations suggesting that EGFR inhibitor-related skin toxicity may serve as a surrogate marker of treatment efficacy. It should also be noted that the incidence of cetuximab-related acneiform rash observed in our study was lower than that reported in most prospective clinical trials. This difference may be attributable to the retrospective design of the study, potential underreporting of low-grade dermatologic toxicities in routine clinical practice, and differences in toxicity assessment and documentation. Therefore, the observed association between dermatologic toxicity and survival should be interpreted with appropriate caution.

Interestingly, both BMI and ALI demonstrated significant associations with survival in univariable analyses but lost significance after multivariable adjustment. Higher BMI was associated with improved OS, a finding that may partially reflect the so-called “obesity paradox”, previously described in several malignancies, where greater nutritional reserve may confer resilience against cancer-related cachexia and treatment-related toxicity [13,14]. Similarly, although ALI integrates nutritional and inflammatory components and has shown prognostic value across different cancer types, its prognostic impact in our cohort may have been attenuated after adjustment for stronger clinicopathological factors such as renal function, treatment response, and primary tumor localization [6].

Several limitations of this study should be acknowledged. First, the retrospective design and single-center nature of the study, together with the relatively small sample size and the heterogeneity of primary tumor sites and treatment approaches, may have introduced selection bias and limited the generalizability of the findings. Second, the relatively small sample size and limited number of events in certain subgroup analyses may have affected statistical precision, particularly in multivariable modeling. Furthermore, because multiple variables were explored in this retrospective analysis, the possibility of multiplicity and chance findings cannot be excluded. Therefore, the identified prognostic factors should be considered hypothesis-generating and require validation in larger prospective studies. Third, treatment approaches were heterogeneous, reflecting routine clinical practice and evolving treatment strategies over the long study period. Moreover, the study period spanned more than 13 years, during which the treatment landscape for recurrent/metastatic HNSCC evolved substantially, particularly following the introduction of immune checkpoint inhibitors into routine clinical practice. These temporal changes may have influenced patient selection, treatment sequencing, and clinical outcomes, thereby contributing to heterogeneity within the study population. In addition, potentially relevant prognostic variables such as HPV/p16 status, PD-L1 expression, and detailed molecular characteristics were not consistently available for all patients. Despite these limitations, our study provides clinically meaningful data regarding prognostic factors and treatment outcomes in cetuximab-treated R/M HNSCC patients.

Despite its limitations, the present study has several strengths. To our knowledge, this study represents a comprehensive evaluation of patients with cetuximab-treated R/M HNSCC, incorporating both clinicopathological and treatment-related prognostic factors. Moreover, the identification of CKD, oral cavity localization, and treatment response as independent determinants of survival may help improve risk stratification and guide individualized treatment planning in routine oncology practice.

## 5. Conclusions

In conclusion, this study demonstrated that cetuximab-based treatment remains an effective therapeutic option in patients with recurrent or metastatic head and neck squamous cell carcinoma (R/M HNSCC), with survival outcomes comparable to previously reported in clinical trials and other retrospective studies. Chronic kidney disease, oral cavity localization of the primary tumor, and progressive disease as the best response to first-line treatment, and the absence of grade 1–2 cetuximab-related dermatologic toxicity emerged as independent prognostic factors for overall survival. These findings may contribute to improved risk stratification and individualized treatment planning in routine oncology practice. Further prospective multicenter studies with larger cohorts are warranted to validate these observations.

## Figures and Tables

**Table 1:** Baseline demographic and clinicopathological characteristics.

Variable	*n*	%
**Sex**		
Female	5	8.8
Male	52	91.2
**Age at diagnosis**		
≤65 years	27	47.4
>65 years	30	52.6
**Body mass index (BMI)**		
≤25 kg/m^2^	32	57.1
>25 kg/m^2^	24	42.9
**ECOG performance status**		
0–1	54	94.7
2	3	5.3
**Comorbidities**		
Chronic kidney disease (CKD)	7	12.3
Chronic obstructive pulmonary disease (COPD)	5	8.8
Diabetes mellitus	9	15.8
Coronary artery disease (CAD)	12	21.1
Hypertension	16	28.1
**Metastatic sites**		
Lung	35	61.4
Liver	6	10.5
Brain	2	3.5
Distant lymph node metastasis	36	63.2
Bone	12	21.1
**Primary tumor site**		
Oropharynx	2	3.5
Hypopharynx	6	10.5
Larynx	29	50.9
Oral cavity	11	19.3
Other	9	15.8
**Tumor presentation**		
Recurrent disease	26	45.6
De novo metastatic disease	31	54.4

Abbreviations: BMI, body mass index; ECOG, Eastern Cooperative Oncology Group; CKD, chronic kidney disease; COPD, chronic obstructive pulmonary disease; CAD, coronary artery disease.

**Table 2:** Treatment characteristics of the study population.

Variable	*n*	%
**Previous history of induction chemotherapy**		
No	51	89.5
Yes	6	10.5
**History of curative radiotherapy**		
No	32	56.1
Yes	25	43.9
**History of surgery**		
No	47	82.5
Yes	10	17.5
**First-line treatment regimen**		
Cetuximab + cisplatin + 5-fluorouracil	29	53.7
Cetuximab + carboplatin + 5-fluorouracil	7	13.0
Other cetuximab-containing regimens	18	33.3
**Best response to first-line treatment**		
Complete response (CR)	5	9.3
Partial response (PR)	16	29.6
Stable disease (SD)	14	25.9
Progressive disease (PD)	19	35.2
**Cetuximab-related grade 1–2 adverse events** (all dermatologic; acneiform rash)		
No	46	80.7
Yes	11	19.3
**Second-line treatment**		
None	34	59.6
Nivolumab	21	36.8
Other	2	3.5

Abbreviations: CR, complete response; PR, partial response; SD, stable disease; PD, progressive disease.

**Table 3:** Kaplan–Meier survival analysis for overall survival according to significant clinicopathological and treatment-related factors.

Variable	Subgroup	*n*	Survivors *n* (%)	Mean OS, Months (95% CI)	Median OS (Months)	*p*
BMI	≤25	30	2 (6.7)	12.4 (9.7–15.1)	11.9	0.002
	>25	23	5 (21.7)	25.7 (16.9–34.6)	19.1	
Chronic kidney disease (CKD)	No	47	6 (12.8)	19.5 (14.6–24.4)	14.6	0.003
	Yes	6	1 (16.7)	6.9 (1.8–11.9)	3.5	
Primary tumor site	Hypopharynx	5	0 (0.0)	16.2 (6.9–25.5)	12.7	0.027
	Larynx	26	3 (11.5)	20.2 (14.8–25.7)	15.7	
	Oral cavity	11	0 (0.0)	9.3 (4.9–13.7)	6.9	
	Other	9	3 (33.3)	12.8 (9.3–16.3)	12.4	
Best response to first-line treatment	CR	5	2 (40.0)	37.7 (14.6–60.7)	23.7	<0.001
	PR	15	2 (13.3)	18.7 (14.7–22.7)	15.7	
	SD	14	2 (14.3)	20.4 (15.4–25.3)	19.1	
	PD	19	1 (5.3)	9.9 (3.7–16.1)	5.0	
Cetuximab-related dermatologic toxicity	No	42	4 (9.5)	13.5 (10.9–16.2)	13.2	<0.001
	Yes (grade 1–2 acneiform rash)	11	3 (27.3)	34.4 (20.3–48.5)	30.4	

Abbreviations: OS, overall survival; CI, confidence interval. Kaplan–Meier survival analysis with log-rank test.

**Table 4:** Univariable and multivariable Cox regression analyses for overall survival.

Variables	Univariable Analysis						Multivariable Analysis					
	B	SE	*p*	HR	95% CI HR		B	SE	*p*	HR	95% CI HR	
					Lower	Upper					Lower	Upper
BMI ≤ 25 kg/m^2^	1.013	0.344	0.003 *	2.755	1.404	5.403	0.523	0.409	0.201	1.688	0.757	3.762
Chronic kidney disease (CKD)	1.353	0.498	0.007 *	3.870	1.459	10.260	1.638	0.567	0.004 *	5.147	1.694	15.642
Primary tumor site (oral cavity)	0.983	0.360	0.006 *	2.672	1.319	5.413	0.935	0.378	0.013 *	2.547	1.215	5340
Best response to first-line treatment (PD)	1.292	0.317	0.000 *	3.640	1.954	6.778	1.491	0.372	0.000 *	4.443	2.143	9212
Absence of grade 1–2 cetuximab-related dermatologic toxicity	1.325	0.419	0.002 *	3.763	1.656	8.550	1.164	0.525	0.027 *	3.204	1.145	8964

Abbreviations: BMI, body mass index; HR, hazard ratio; CI, confidence interval; SE, standard error; PD, progressive disease; * *p* < 0.05 indicates statistical significance.

## Data Availability

The data presented in this study are available on reasonable request from the corresponding author. The data are not publicly available due to patient privacy and ethical restrictions.
